# Insecticide resistance profile of the *Anopheles gambiae* s.l. mosquitoes in Nigeria: a 5-year malaria vector surveillance report

**DOI:** 10.1186/s12936-026-06083-9

**Published:** 2026-08-10

**Authors:** Adedapo O. Adeogun, Ayodele S. Babalola, Tolulope A. Oyeniyi, Okoko O. Oyale, Romoke T. Izekor, Oluwakemi Adetunji, Abiodun Olakiigbe, Olalekan Olagundoye, Julius A. Bamidele, Adetunji I. Adeniran, Nofisat F. Lawal, Philemon Babylon, Atinga Atimi, Sufi A. Danjuma, Tasiu Shuaibu, Jibrin M. Mabu, Kabiru Salisu, Asma’u H. Abubakar, Mahmud Sufyan, Kabir B. Abdullahi, Adamu Isa, Ezra Abba, Abdulmalik B. Shuaibu, Victor N. Enwemiwe, Esiwo Eric, Wama B. Emmanuel, Liatu C. Garba, Abdullahi M. Daskum, Monsuru A. Adeleke, Chioma C. Ojianwuna, Elkanah O. Sambo, Adedayo Oduola, Petrus U. Inyama, Lazarus Samdi, Abiodun Obembe, Mustapha M. Dogara, Kennedy P. Yoriyo, Suleiman Mohammed, Rebecca S. Naphtali, Chioma Amajoh, Adesoye Oluwaseun, Fatima Ali, Adesola Musa, Wahedi Jasini, Bala Mohammed, Esema Mary, Omo-Eboh Mamudu, Olufunmilayo A. Idowu, Adeolu Ande, Kayode I. Olayemi, Manu Yayo Abdulsalami, Ademu Cyril, Okoronko Chukwu, Ozor Lynda, Ssekitooleko James, Kawu Issa, Mokuolu Olugbenga, Nnenna Ogbulafor, Awolola Samson

**Affiliations:** 1 Nigerian Institute of Medical Research, Yaba, Lagos, Nigeria; 2 National Malaria Elimination Program, Federal Ministry of Health, Abuja, Nigeria; 3 Federal University of Agriculture, Abeokuta, Nigeria; 4 Taraba State University, Jalingo, Nigeria; 5 State College of Health Science, Takum, Adamawa Nigeria; 6 Department of Biological Sciences, Faculty of Science, Federal University, Dutse, Jigawa Nigeria; 7 Umar Suleiman College of Education, Gashu’a, Yobe State Nigeria; 8 Yobe State University, Damaturu, Nigeria; 9 University of Science and Technology, Kano, Kano State Nigeria; 10 Yusuf Maitama Sule University, Kano, Kano State Nigeria; 11 Department of Biology, Umaru Musa Yar’adua University, Batagarawa, Katsina State Nigeria; 12 Federal University Dutsinma, Dutsinma, Katsina Nigeria; 13 Department of Zoology, Faculty of Science, Gombe State University, Gombe, Nigeria; 14 Department of Animal & Environmental Biology, Delta State University, Abraka, Delta State Nigeria; 15 Department of Biological Sciences, Taraba State University, Jalingo, Nigeria; 16 Department of Zoology, Faculty of Basic and Applied Sciences, Osun State University, Osogbo, Nigeria; 17 ABT Associates, Keffi, Nasarawa State Nigeria; 18 Department of Zoology, Kwara State University, Malete, Kwara Nigeria; 19 Department of Zoology, Madibbo Adama University of Technology, Yola, Adamawa State Nigeria; 20 Community Vision, Abuja, Nigeria; 21 University of Abuja, Abuja, FCT Nigeria; 22 Department of Zoology, Adamawa State University, Mubi, Adamawa State Nigeria; 23 Department of Zoology, University of Ilorin, Ilorin, Kwara State Nigeria; 24 Department of Animal Biology, Federal University of Technology, Minna, Nigeria; 25 Centre for Infectious Diseases Research, Bayero University, Kano, Nigeria; 26 World Health Organization, Abuja, Lagos State Nigeria; 27 Global Fund, Abuja, Federal Capital Territory Nigeria; 28 Faculty of Health Sciences, College of Health Sciences, University of Ilorin, Ilorin, Nigeria

**Keywords:** *Anopheles gambiae s.l.*, Insecticide resistance, Pyrethroid, *kdr*-w, Nigeria, West-Africa

## Abstract

**Background:**

Nigeria bears the highest malaria burden globally, with vector control strategies relying heavily on the use of Long-lasting Insecticidal nets (LLINs). However, the widespread emergence of insecticide resistance threatens the effectiveness of this intervention in the country. This study provides a comprehensive 5-year overview (2020–2024) of the resistance status and resistance mechanisms of *Anopheles* mosquitoes in Nigeria.

**Methods:**

Immature stages of mosquitoes were collected from 72 local government areas (spanning 12 States), raised to adults and exposed to standard concentrations of permethrin, deltamethrin, alphacypermethrin, and pirimiphos-methyl insecticide papers according to WHO criteria for 5 years (2020–2024). Furthermore, molecular test and bioassays were used to determine the involvement of kdr-L995F mutation and oxidase-based metabolic enzymes in areas where resistance was confirmed. All mosquitoes tested were identified morphologically. Spatial and temporal patterns of insecticide susceptibility results were visualised using geospatial mapping tools in R (sf, tidyr, ggplot2).

**Results:**

Members of the *Anopheles gambiae* complex accounted for 95% of all mosquitoes tested across the 72 LGAs, with *Anopheles coluzzii* comprising 79.8% of collections. Other species encountered included *An. funestus, An. pretoriensis, An. malculipalpis,* and *An. nilli*. Widespread and persistent resistance to pyrethroid insecticides was detected throughout the study period. Mortality to permethrin remained consistently low (0–75%), indicating high levels of resistance across most locations. Resistance to deltamethrin and alphacypermethrin was also widespread but generally less pronounced, with mortality ranging from 10 to 90% and 20–80%, respectively. Temporal trends revealed localized reductions in resistance intensity between 2022 and 2023, coinciding with periods of large-scale LLINs distribution in several States. Synergist bioassays demonstrated substantial involvement of oxidase-based metabolic mechanisms, suggesting that multiple resistance mechanisms are operating across Nigeria. The frequency of the kdr-w mutation ranged from 0.0 to 0.7 and generally increased between 2021 and 2023 before declining slightly in 2024. While kdr-w frequency was significantly associated with reduced mortality to deltamethrin (τ =  − 0.179, p < 0.001), no corresponding association was observed for permethrin (τ ≈ 0, p = 0.998), and the mutation was not a significant predictor of pyrethroid mortality overall after accounting for insecticide type and spatial clustering (β = 10.50, p = 0.078). These findings indicate that the contribution of kdr-mediated resistance varies among pyrethroid compounds.

**Conclusion:**

The predominance of Anopheles *coluzzii* in tested collections, coupled with pervasive pyrethroid resistance and widespread oxidase-based metabolic resistant mechanisms, poses significant challenges to malaria vector control efforts in Nigeria. These findings underscore the urgent need to prioritize efforts at managing metabolic resistance mechanisms through integrated vector control and further strengthen IRS with the use of non-pyrethroid insecticides. Enhanced surveillance, including molecular diagnostics and monitoring of agricultural pesticide use, will be critical for sustaining malaria control gains and informing adaptive, evidence-driven intervention planning in Nigeria.

**Supplementary Information:**

The online version contains supplementary material available at https://doi.org/10.1186/s12936-026-06083-9.

## Background

Malaria remains one of the most significant public health challenge in Nigeria, contributing an estimated 27% of global malaria cases and 32% of global malaria deaths, the highest of any country worldwide [1]. Transmission is sustained predominantly by members of the *Anopheles gambiae* complex, including *An. coluzzii*, *An. gambiae* and *An. arabiensis* [2, 3]. The behavioural and ecological differences among members of the *Anopheles gambiae* complex influence malaria transmission dynamics and may affect the operational performance of vector control interventions [2, 3]. Understanding changes in the relative abundance of these sibling species therefore provides important ecological context for interpreting nationwide insecticide resistance surveillance. Despite large-scale implementation of vector control interventions, malaria prevalence in many regions of Nigeria remains high, emphasizing the need for continuous vector surveillance and insecticide resistance profiling.

Long-lasting insecticidal nets (LLINs) remain the core of malaria prevention in Nigeria [4, 5]. Most LLIN distributed in the country are pyrethroid-based, with the National Malaria Elimination Programme introducing alternative insecticide laden nets in areas of pyrethroid resistance based on emerging research evidence. Other types of vector control are considered but less implemented. However, the increasing spread and intensity of insecticide resistance among *Anopheles* vectors jeopardizes the effectiveness of pyrethroid-based interventions. Across sub-Saharan Africa, pyrethroid resistance, driven by target-site mutations (e.g., *kdr* L995F/S) and metabolic detoxification mechanisms (including elevated cytochrome P450 monooxygenases, GSTs, and esterases) has become widespread [6–8]. These mechanisms reduce mortality following LLIN exposure and are associated with reduced epidemiological impact of nets where resistance intensity is high [8].

In the country, several location-specific studies have documented pyrethroid resistance, particularly in the northern and southern agricultural belts [9–11]. Most of these prior studies were limited in geographic scope and time frame, leaving considerable uncertainty regarding national trends and changes over time. The expansion of agriculture, especially rice irrigation schemes and year-round vegetable farming, has further intensified selection pressure from pyrethroid and organophosphate insecticides, contributing to cross-resistance between public-health and agricultural insecticides [12–14]. Yet, systematic multi-year surveillance across multiple ecological zones has been lacking. To date, no study has provided a coordinated longitudinal assessment of insecticide resistance spanning multiple years and ecological zones at a national scale in Nigeria. Consequently, important questions remain regarding the temporal stability of resistance patterns, the distribution of resistance mechanisms, and whether resistance profiles differ across the country’s diverse ecological settings.

Members of the *Anopheles gambiae* complex differ in larval ecology, host preference, and vectorial capacity [3]. For example, *An*. *coluzzii* tends to exploit permanent or semi-permanent water bodies, including anthropogenic breeding sites, making it more adapted to expanding irrigation and urbanization. By contrast, *An*. *gambiae* s.s. typically breeds in temporary rain-dependent pools, while *An*. *arabiensis* exhibits more exophagic and zoophilic tendencies [2, 3, 15, 16]. Shifts in species dominance can therefore alter indoor versus outdoor transmission patterns, influence net efficacy, and drive operational decision-making. Emerging evidence from West Africa suggests a rising dominance of *An*. *coluzzii* in certain settings, potentially linked to environmental change and adaptive resilience [2, 3, 17]. The World Health Organization (WHO) recommends continuous insecticide resistance monitoring, including phenotypic assays and synergist tests to support insecticide resistance management (IRM) plans [18].

This study therefore addresses the lack of coordinated longitudinal insecticide resistance surveillance across Nigeria by providing the first 5-year (2020–2024) nationwide assessment of malaria vector species composition, insecticide susceptibility, and resistance mechanisms spanning the country's principal ecological zones. We hypothesized that pyrethroid resistance would be widespread but spatially heterogeneous, with metabolic resistance mechanisms contributing more substantially than target-site (kdr) mutations to the observed phenotypic resistance. Specifically, the study aimed to (i) characterize temporal changes in the composition of the *Anopheles* *gambiae* complex, (ii) assess trends in susceptibility to commonly used public-health insecticides, (iii) determine the distribution of target-site and metabolic resistance mechanisms, and (iv) evaluate the relationship between kdr-w frequency and phenotypic resistance. By integrating longitudinal phenotypic, molecular and synergist data, the study provides evidence to inform insecticide resistance management and malaria vector control strategies in Nigeria.

## Methods

### Study area

This study was conducted across 12 states in Nigeria (9.0820° N, 8.6753° E). Nigeria is characterized by diverse ecological zones ranging from mangrove and rainforest ecosystems in the south to Guinea, Sudan, and Sahel savannahs in the north. The study states collectively span these major ecological zones and encompass a wide range of climatic conditions, with annual temperatures ranging from approximately 24–30 °C and annual rainfall ranging from about 300–1,900 mm. Malaria transmission occurs throughout the country but varies in intensity across ecological zones, with vector control relying predominantly on long-lasting insecticidal nets (LLINs) distributed through routine and mass campaign programmes.

**Fig. 1:**
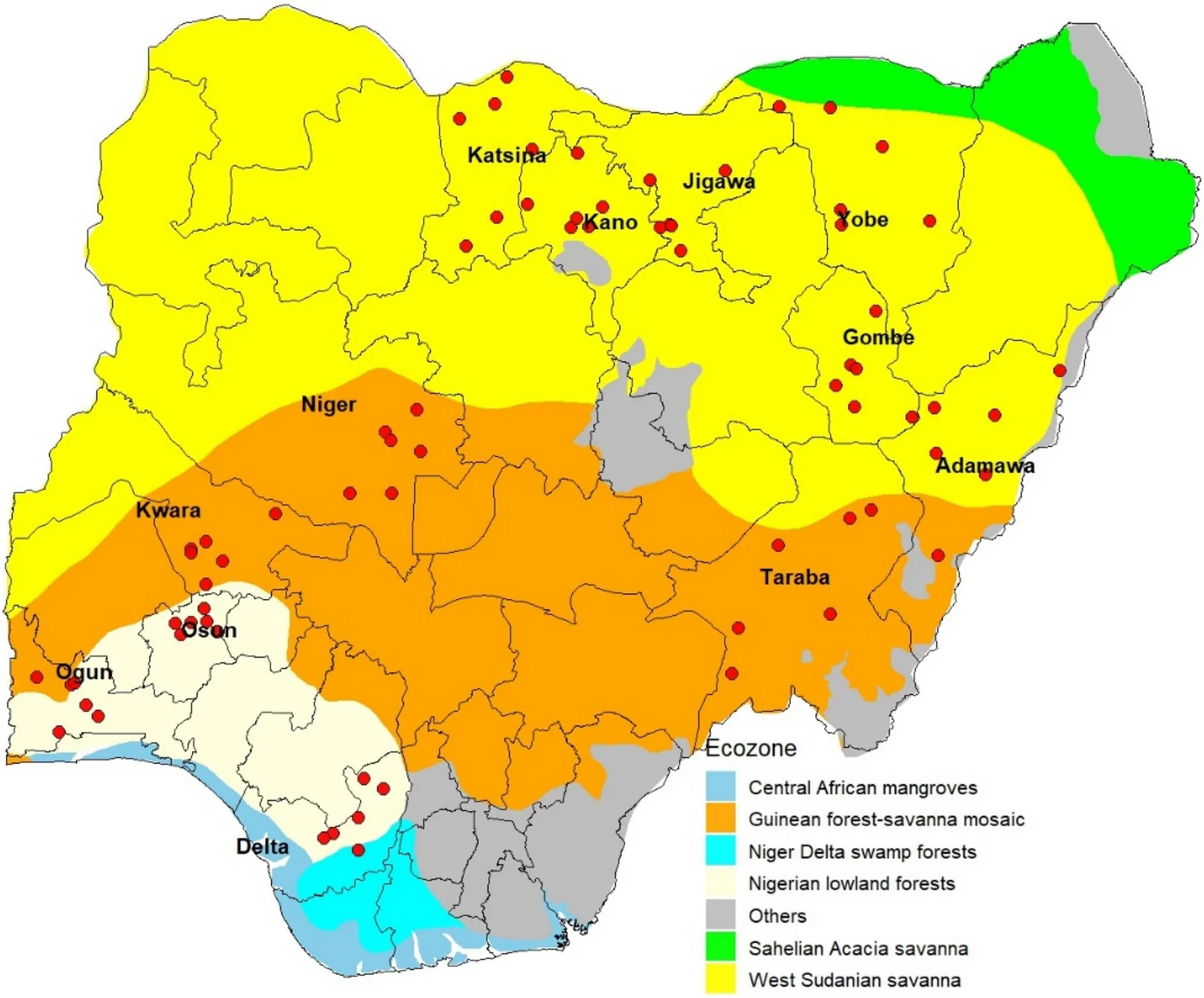
Study area across the major ecozones of Nigeria, with red dots representing the LGAs where mosquito samples were collected

Six Local Government Areas (LGAs) were surveyed in each state, covering a total of 72 LGAs and encompassing all ecological zones within the country. The 12 states were selected as part of the National Malaria Elimination Programme's sentinel surveillance network to provide coverage across Nigeria's major ecological zones, including rainforest, mangrove, Guinea savannah, Sudan savannah, and Sahel savannah ecosystems. Within each state, six LGAs were selected in consultation with state malaria programmes to provide broad geographic representation and capture variation in ecological conditions and malaria transmission settings (Fig. 1).

A cross-sectional study design was employed, with at least five locations selected within each LGA for mosquito larval collection. Additional details regarding ecological characteristics of the surveillance sites have been published previously [19].

### Mosquito collection and identification

Mosquito larvae were collected over a 5-year period (2020–2024) using the World Health Organization (WHO) entomological protocols for field and laboratory studies [18]. Breeding sites were identified by trained entomologists and surveyed using a standardized protocol across all study locations. Sampling was conducted repeatedly during the rainy season (April–October) of each year, corresponding to the peak malaria transmission season in Nigeria, when larval habitats and *Anopheles gambiae* s.l. populations are most abundant and national vector surveillance is routinely undertaken. Larvae were collected from a range of natural and human-made breeding habitats, including rice fields (predominantly in northern Nigeria), rain-fed puddles of varying sizes, stagnant water associated with drainage channels, tyre tracks, borrow pits, and other temporary water bodies. Within each LGA, at least five sampling locations were surveyed, and larval collections were undertaken proportionally to breeding-site size (based on surface area and water volume) to ensure comparable sampling effort across habitats and study sites. Although the relative abundance of sibling species may vary seasonally in response to ecological and climatic conditions, conducting collections during the same transmission season each year ensured methodological consistency and enabled robust comparisons of temporal trends in species composition and insecticide resistance across the surveillance period.

The locations of all breeding sites were georeferenced using a GPS device. Sampling was conducted proportionally to the size of each breeding site, ensuring adequate representation of larger sites. Collected larvae were transported to the insectary. Anopheline larvae were first morphologically identified and separated from non-anopheline species. The larvae were reared to adulthood under ambient laboratory conditions, and the emerged adults were identified morphologically using the identification keys of Coetzee [20], with a × 1000 Dino-Lite HD color CMOS sensor high-speed digital microscope (model number AD4113T-12 V). Mosquitoes belonging to the *Anopheles* *gambiae* complex (*An. gambiae* s.l.) were further identified to species level using species-specific PCR assays described by Scott et al. [21], allowing discrimination of the principal vector species within the complex.

Over the 5-year surveillance period, approximately 144,000 adult mosquitoes were processed for insecticide susceptibility testing. From these, approximately 32,000 mosquitoes exposed to the insecticide bioassays were randomly selected for molecular characterization, irrespective of their phenotypic outcome (dead or surviving). Molecular analyses were performed using different body parts of the same mosquito, with one body part used for species identification PCR and another for *kdr-w (L995F*) genotyping. Species identification was successfully completed for 30,311 specimens, corresponding to a PCR amplification success rate of 94.7%. Species composition and *kdr-w* estimates presented in this study are based on successfully amplified samples only.

### Insecticide resistance monitoring

The WHO tube bioassay method was employed to test mosquitoes against diagnostic concentrations of insecticides. The diagnostic concentrations of insecticide-impregnated papers supplied through WHO-approved procurement channels and produced by the Vector Control Research Unit (VCRU), Universiti Sains Malaysia, Penang, Malaysia. Throughout the surveillance period, standard WHO quality assurance procedures were followed, including the use of concurrent control exposures and adherence to WHO-recommended storage, handling, and testing protocols. Mortality in control groups was assessed according to WHO criteria.

Adult mosquitoes aged 2–5 days (25 mosquitoes per replicate, in 4 replicates) were exposed to WHO-approved insecticide-impregnated papers containing the following active ingredients: Permethrin (0.75%), Deltamethrin (0.05%), Alphacypermethrin (0.05%), and Pirimiphos-methyl (0.5%) for one hour. The assays were conducted under ambient conditions at 27 ± 2 °C and 75 ± 10% relative humidity.

Following the one-hour exposure, mosquitoes were transferred back into holding tubes and provided with 10% sugar solution for 24 h, after which mortality was recorded. A control group was included in each test, where mosquitoes were exposed to untreated papers under the same conditions. If mortality in the control group ranged between 5 and 20%, Abbott’s formula was applied to correct the observed mortality rates. Tests were discarded and repeated if control mortality exceeded 20%.

### Resistance mechanism detection

We further conducted synergist bioassays on mosquito populations that exhibited either possible or confirmed resistance to permethrin, in order to gain insight into the underlying resistance mechanisms. Adult mosquitoes aged 2–5 days (25 per replicate, with 4 replicates) were first exposed to PBO (4%) for 1 h, followed by exposure to the insecticide for another 1 h. Mortality was then recorded after 24 h, in accordance with WHO guidelines [18]. PBO synergist bioassays were performed using permethrin as the representative pyrethroid after preliminary testing demonstrated comparable synergist responses across the three pyrethroids for three consecutive years. Furthermore, PCR assays were conducted to detect the West African knockdown resistance mutation, *kdr-w (L995F)*, using the protocol of Martinez-Torres et al. [22]. This mutation was selected because it is the most widely reported target-site resistance mechanism among members of the *Anopheles gambiae* complex in West Africa, including Nigeria. Other target-site mutations, such as kdr-e (L995S), were not assessed in the present study.

### Data analysis

Resistance status was determined based on 24-h post-exposure mortality, as follows: *Susceptible* if mortality was ≥ 98%, *Possible resistance* if mortality was between 90 and 97%, and *Confirmed resistance* if mortality was < 90% [18]. The results of the synergist assays were interpreted as follows:

- No restoration of susceptibility (Metabolic enzymes not involved): If the mortality rate of mosquitoes exposed to the synergist–insecticide combination is not at least 10% higher than that of mosquitoes exposed to the insecticide alone, it indicates that the synergist did not restore phenotypic susceptibility. This suggests that metabolic enzymes are not involved in the resistance mechanism.
- Partial restoration of susceptibility (Metabolic enzymes partially involved): If the mortality rate of mosquitoes exposed to the synergist–insecticide combination is at least 10% higher than that of mosquitoes exposed to the insecticide alone but remains below 98%, it indicates partial involvement of metabolic enzymes, suggesting that a metabolic mechanism of resistance is present.
- Full restoration of susceptibility (Metabolic enzymes fully involved): If the mortality rate of mosquitoes exposed to the synergist–insecticide combination is ≥ 98% and at least 10% higher than that of mosquitoes exposed to the insecticide alone, it implies full involvement of metabolic enzymes in the resistance mechanism [18].

Spatial and temporal patterns of resistance status, *kdr-w* mutation frequency were analyzed using geo-referenced data collected across selected Nigerian states from 2020 to 2024. Annual resistance status and *kdr-w* frequencies were mapped by location, and a 5-year average was calculated to summarize long-term patterns. The same sentinel locations were surveyed annually from 2020 to 2024. For each location, the 5-year kdr-w mutation frequency were calculated as the arithmetic mean of the annual measurements obtained across the surveillance period. Frequencies were grouped into predefined classes to facilitate visual comparison and identification of spatial clustering.

Associations between *kdr-w* frequency and mosquito mortality following exposure to WHO diagnostic concentrations of pyrethroid insecticides were assessed using Kendall’s tau correlation, selected to account for non-normality and tied values. Correlation analyses were performed separately for permethrin, deltamethrin, and alphacypermethrin. To account for spatial clustering and hierarchical data structure, linear mixed-effects models were fitted with mosquito mortality as the outcome variable. Survey year and insecticide type was included as a fixed effect to account for temporal variation in mosquito mortality across the 5-year surveillance period, while State and LGA (nested within State) were included as random intercepts to account for the hierarchical sampling design. Models were estimated using restricted maximum likelihood, with statistical significance assessed using Satterthwaite’s approximation. Model performance was evaluated using marginal and conditional R^2^ values to quantify variance explained by fixed effects alone and by combined fixed and random effects. All analyses and visualizations were conducted in R (version 4.4).

## Results

### Trend in species composition over a 5-year period (2020–2024) in Nigeria

Table 1 presents the species-level composition of *Anopheles* mosquitoes identified across all study sites, stratified by state. While the initial species composition has been reported in earlier studies, the present analysis reveals a steady annual increase in the population of *Anopheles coluzzii*, alongside a temporal decline in *Anopheles gambiae* and *Anopheles arabiensis*. Overall, Table 1 indicates that *Anopheles coluzzii* was the predominant species, followed by *Anopheles gambiae* and then *Anopheles arabiensis*. This pattern of species composition became increasingly consistent over the study period.

**Table 1:** Species composition of the members of *Anopheles gambiae* s.l complex found in the total mosquitoes collected between 2020 and 2024

Year	Total *Anopheles gambiae* mosquitoes recorded	Number of *Anopheles gambiae* complex species found (%)		
		*Anopheles coluzzii*	*Anopheles gambiae*	*Anopheles arabiensis*
2020	5,304	3,068 (57.8)	1,935 (36.5)	301 (5.7)
2021	5,712	4,653 (81.5)	895 (15.7)	164 (2.9)
2022	5,582	3,881 (69.5)	1,466 (26.3)	235 (4.2)
2023	6,820	5,968 (87.5)	700 (10.3)	152 (2.2)
2024	6,893	6,625 (96.1)	192 (2.8)	76 (1.1)
TOTAL	30,311	24,195 (79.8)	5,188 (17.1)	928 (3.1)

### Susceptibility status of mosquitoes exposed to WHO diagnostic concentrations of Permethrin, Deltamethrin, Alphacypermethrin, and Pirimiphos-methyl over a 5-year period (2020–2024)

**Fig. 2:**
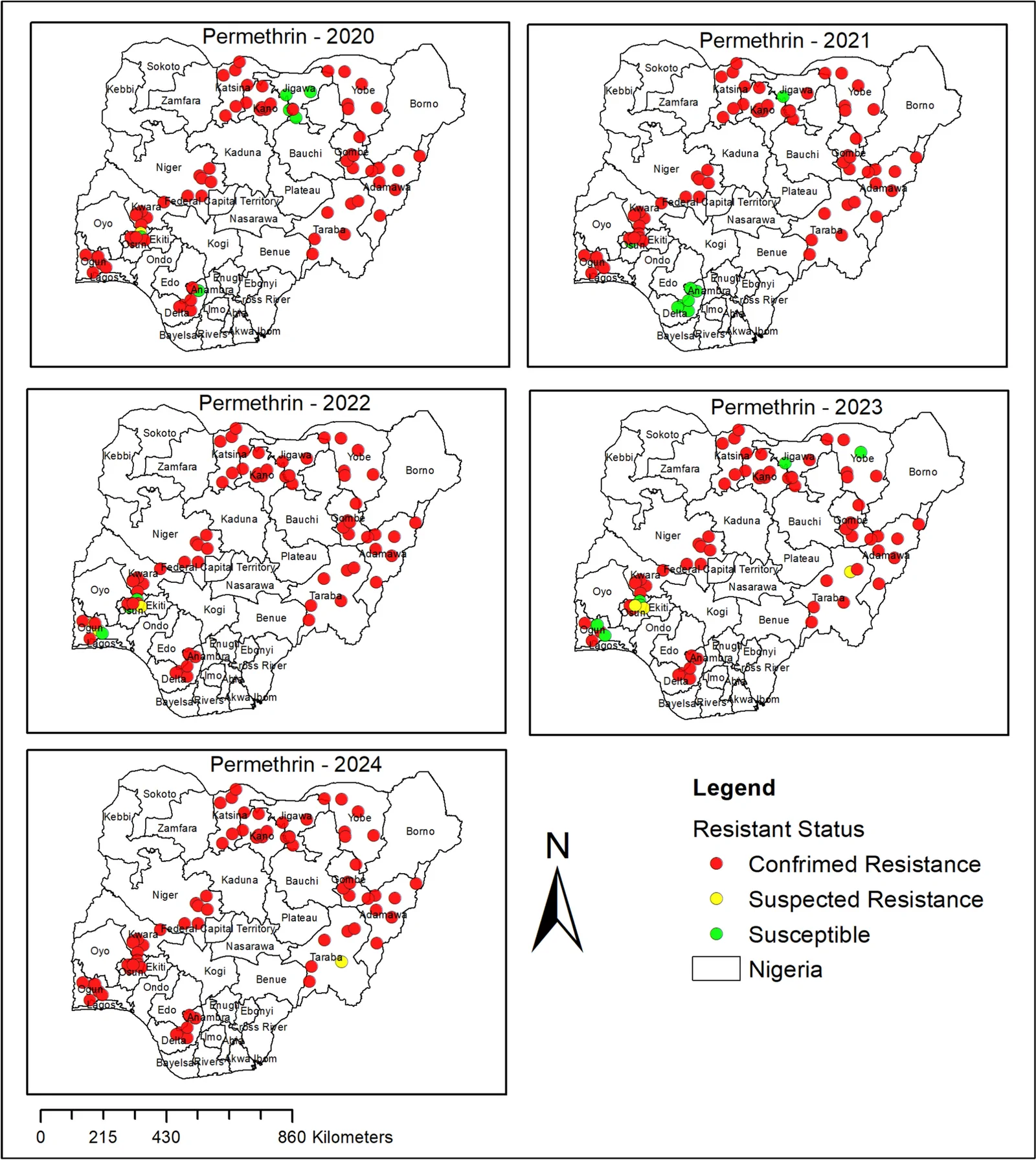
Resistance status of *Anopheles gambiae* s.l. to the diagnostic concentration of permethrin (WHO tube bioassay) in 72 Local Government Areas across 12 states in Nigeria from 2020 to 2024

**Fig. 3:**
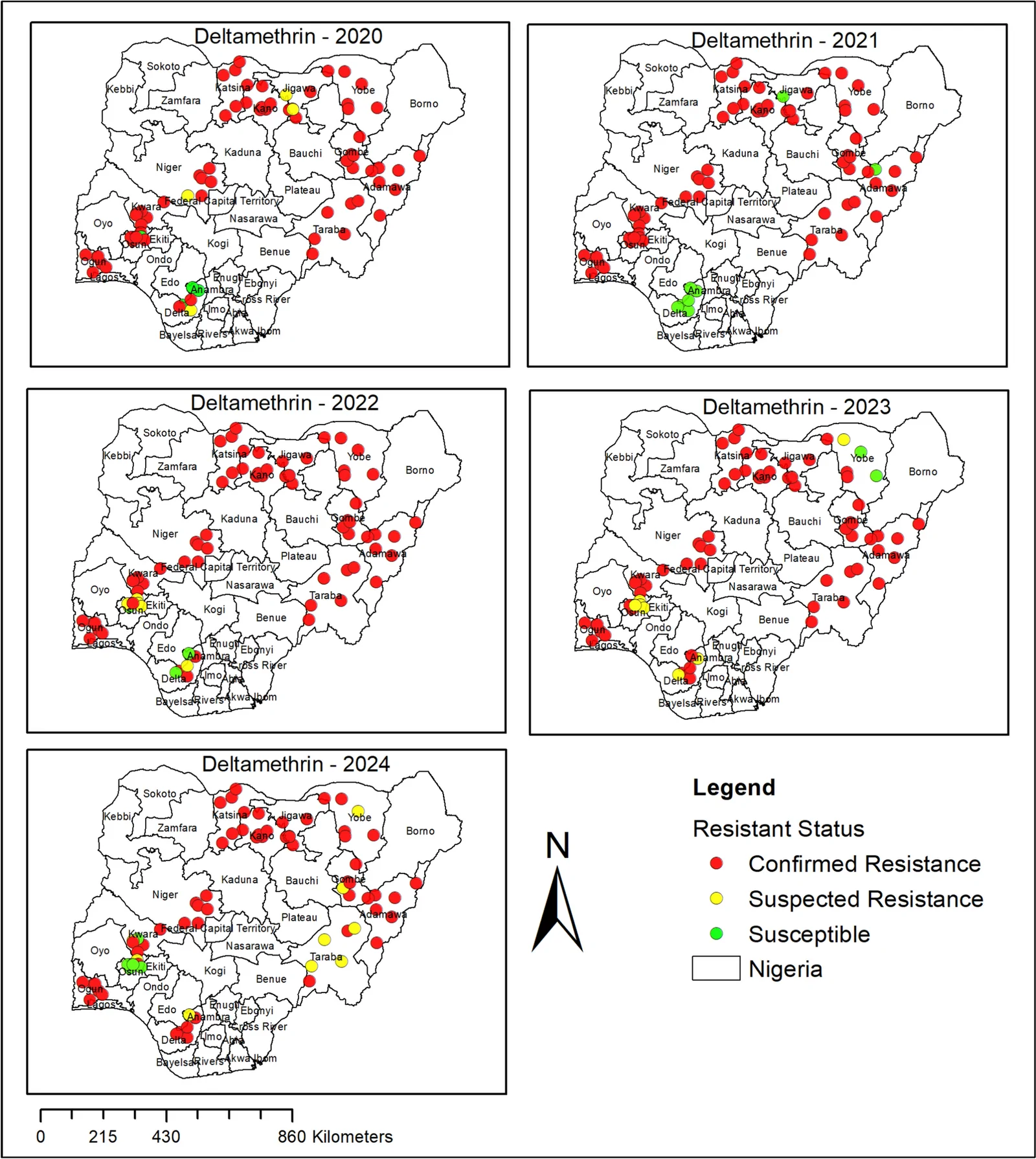
Resistance status of *Anopheles gambiae* s.l. to the diagnostic concentration of deltamethrin (WHO tube bioassay) in 72 Local Government Areas across 12 states in Nigeria from 2020 to 2024

**Fig. 4:**
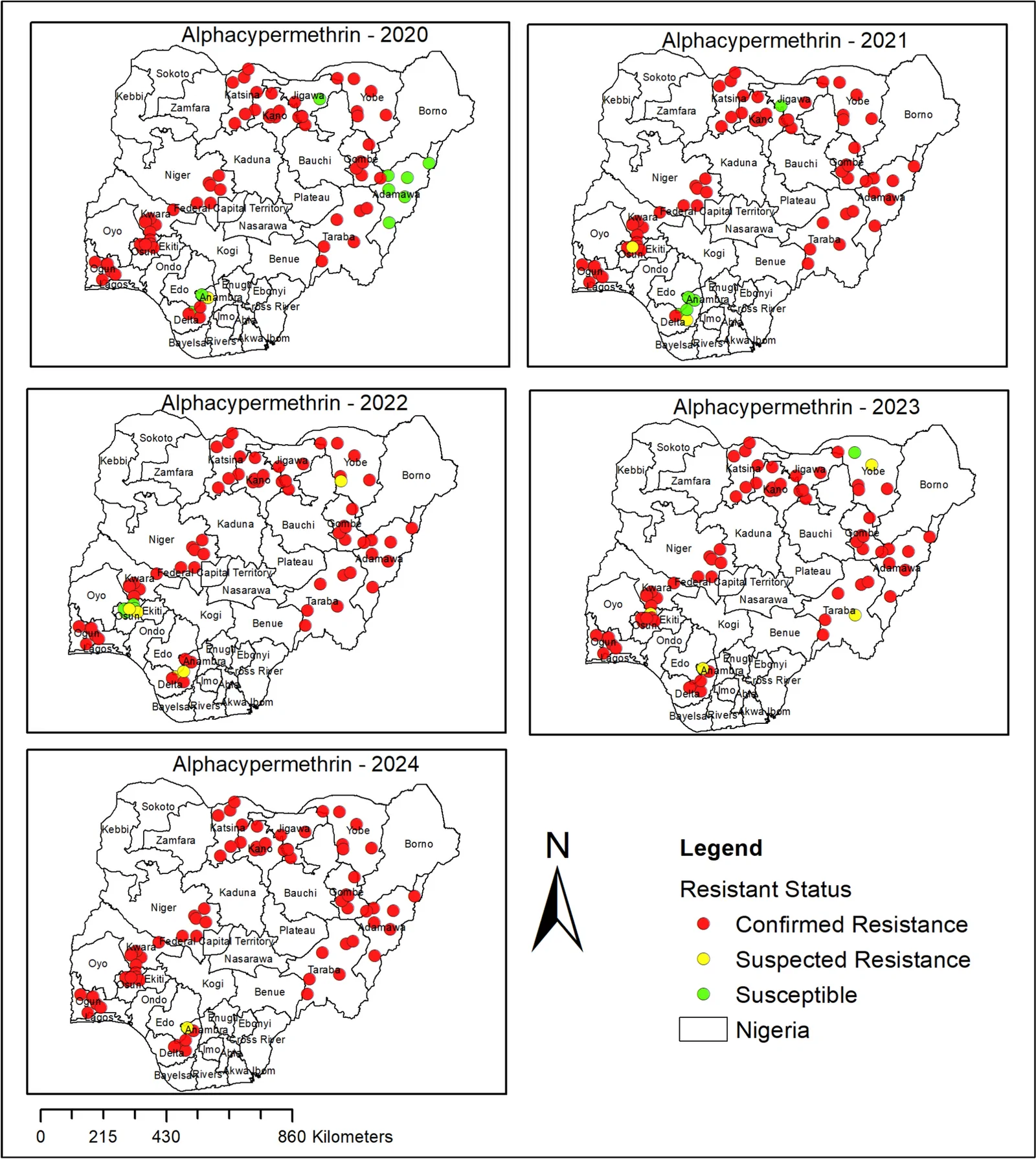
Resistance status of *Anopheles gambiae* s.l. to the diagnostic concentration of alphacypermethrin (WHO tube bioassay) in 72 Local Government Areas across 12 states in Nigeria from 2020 to 2024

**Fig. 5:**
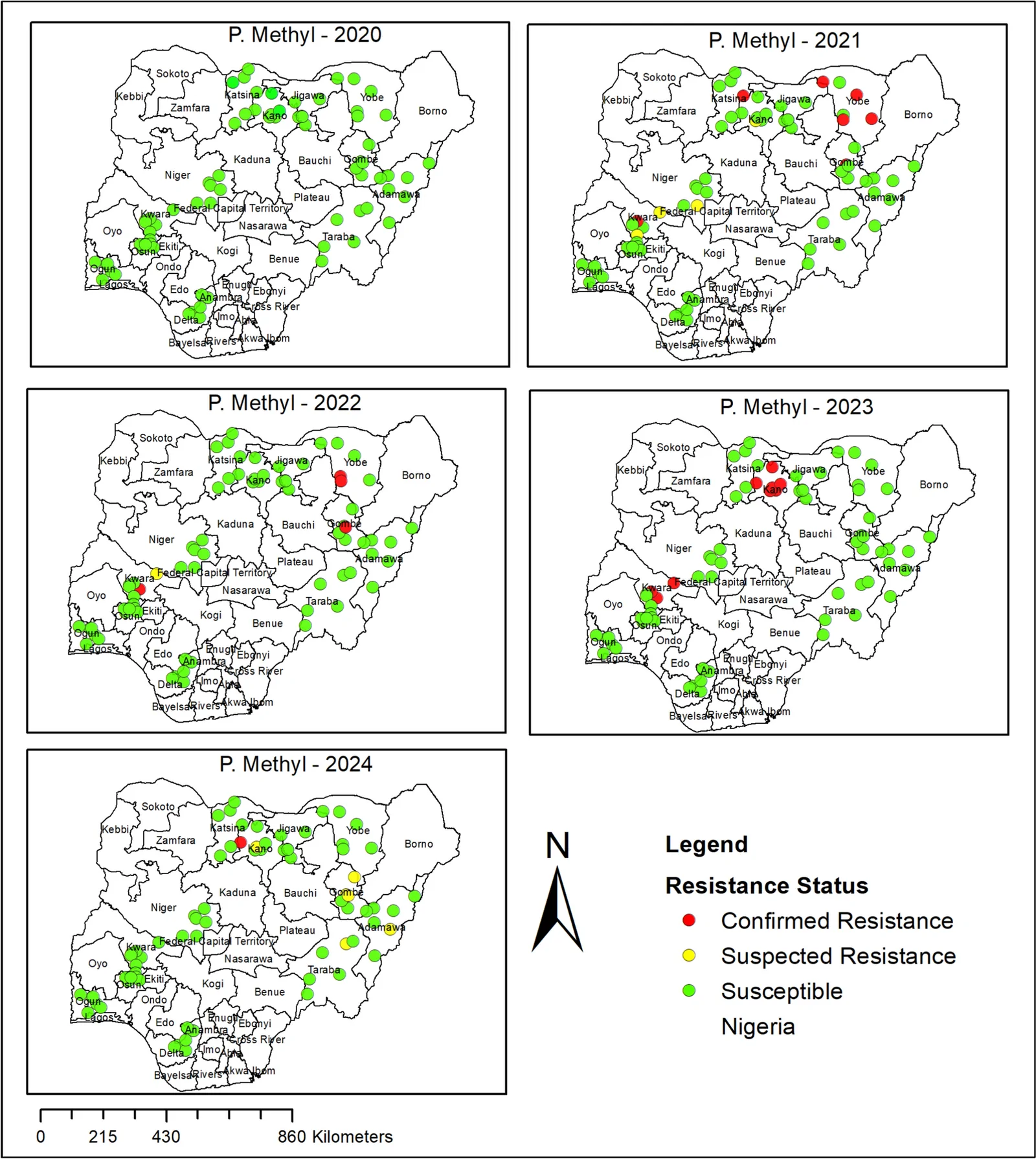
Resistance status of *Anopheles gambiae* s.l. to the diagnostic concentration of pirimiphos-methyl (WHO tube bioassay) in 72 Local Government Areas across 12 states in Nigeria from 2020 to 2024

Malaria vectors exhibited contrasting responses to pyrethroids and pirimiphos-methyl over the 5-year surveillance period. Resistance to the three pyrethroids (permethrin, deltamethrin and alphacypermethrin) was widespread across the 12 surveyed states, whereas susceptibility to pirimiphos-methyl remained high in most locations (Figs. 2, 3, 4, 5). Across the surveillance period, mortality following pyrethroid exposure remained predominantly below 50%, although temporal trends differed among compounds. Permethrin consistently produced the lowest mortality with little evidence of recovery between 2020 and 2024, whereas deltamethrin showed a gradual increase in mortality, reflected by more LGAs shifting from the 0–25% to the 50–75% mortality class by 2024. Mortality following alphacypermethrin remained comparatively stable over time, with only localized improvements. By contrast, pirimiphos-methyl maintained near-complete mortality across most surveyed LGAs throughout the study period, with only localized and transient declines between 2021 and 2023 before returning to uniformly high mortality in 2024 (Supplementary File S1, Figures A–D).

### Determination of the involvement of metabolic resistance mechanisms in the mosquito populations

**Fig. 6:**
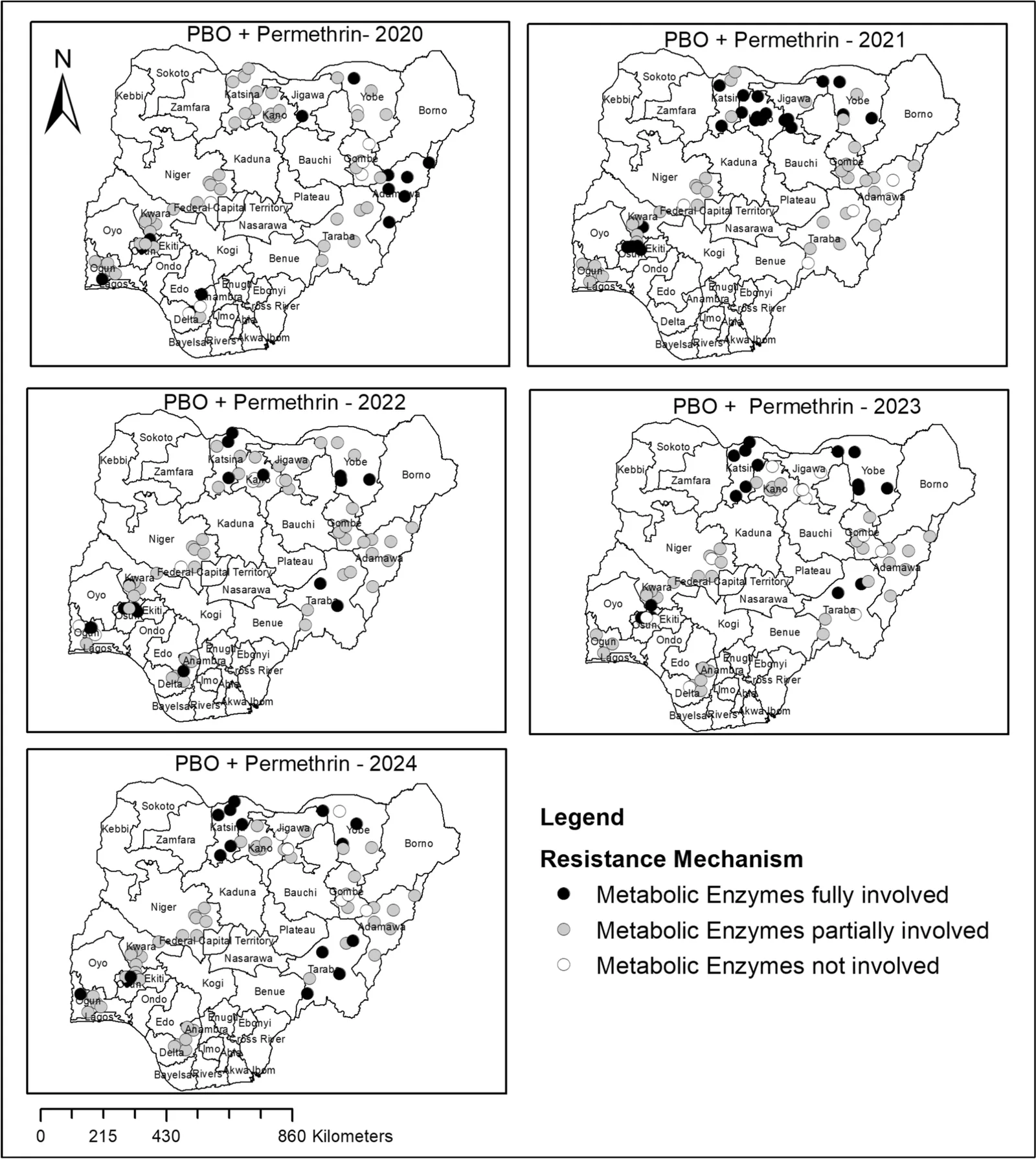
Mechanisms of resistance in *Anopheles gambiae* s.l. against permethrin in 72 Local Government Areas across 12 states in Nigeria from 2020 to 2024, based on interpretations from the synergist bioassay

**Fig. 7:**
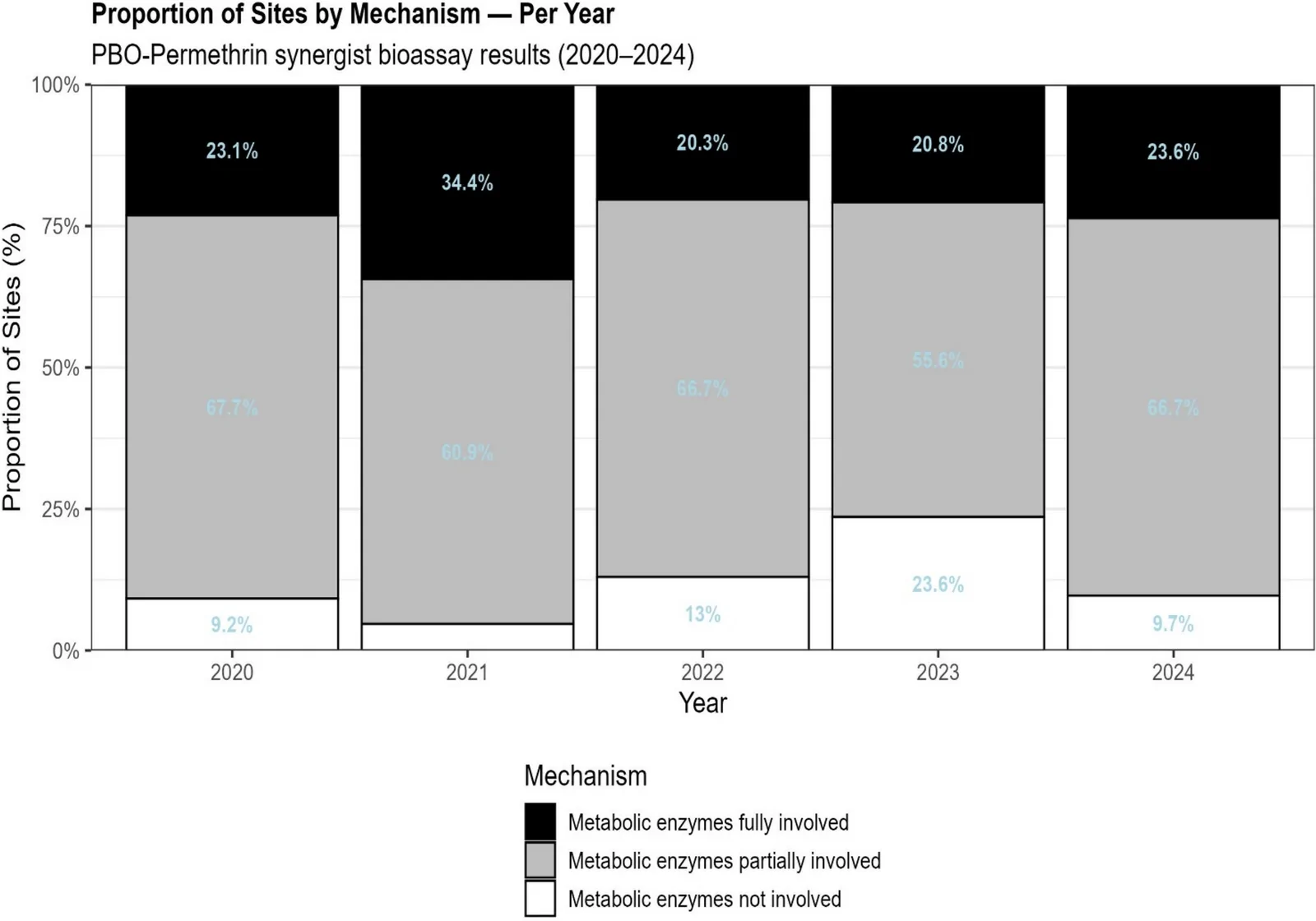
Temporal trends of resistance mechanism in *An. gambiae* s.l. in Nigeria from 2020 to 2024 based on PBO–permethrin synergist bioassay results

The PBO–permethrin synergist bioassays demonstrated that metabolic resistance mechanisms contributed to pyrethroid resistance in the majority of mosquito populations across the 5-year surveillance period (Fig. 6). Across all surveyed sites, metabolic enzymes were either partially or fully involved in 76.4–95.0% of populations annually, whereas no evidence of metabolic enzyme involvement was observed in only 5.0–23.6% of sites. Partial restoration of susceptibility predominated throughout the study period, accounting for 55.6–67.7% of sites annually, while complete restoration ranged from 20.3 to 34.4%, peaking in 2021 before declining modestly in subsequent years (Fig. 7).

**Fig. 8:**
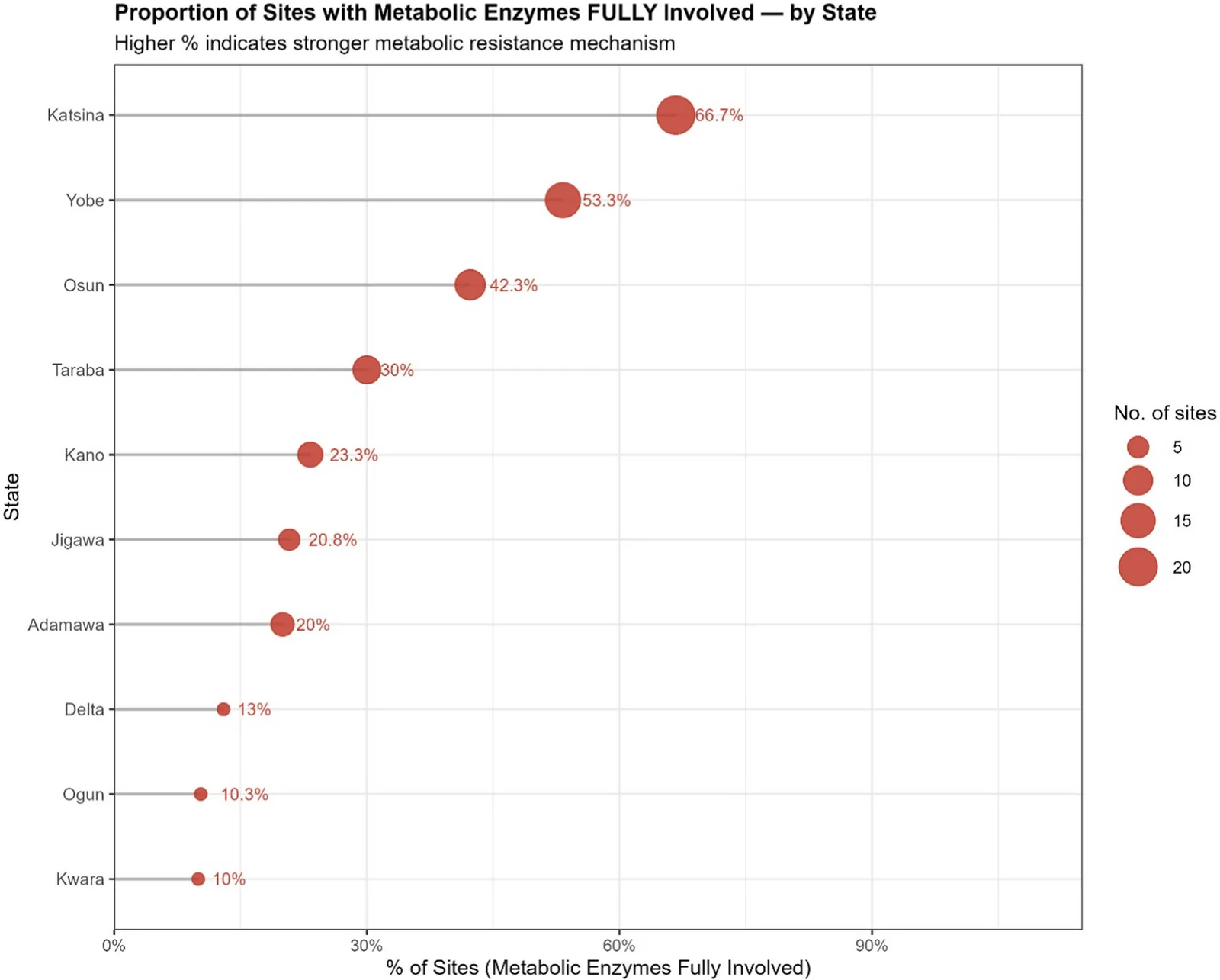
Overall contribution of metabolic resistance mechanisms in *Anopheles* *gambiae* s.l. across the 12 States of Nigeria, 2020–2024, based on PBO–permethrin synergist bioassay results

**Fig. 9:**
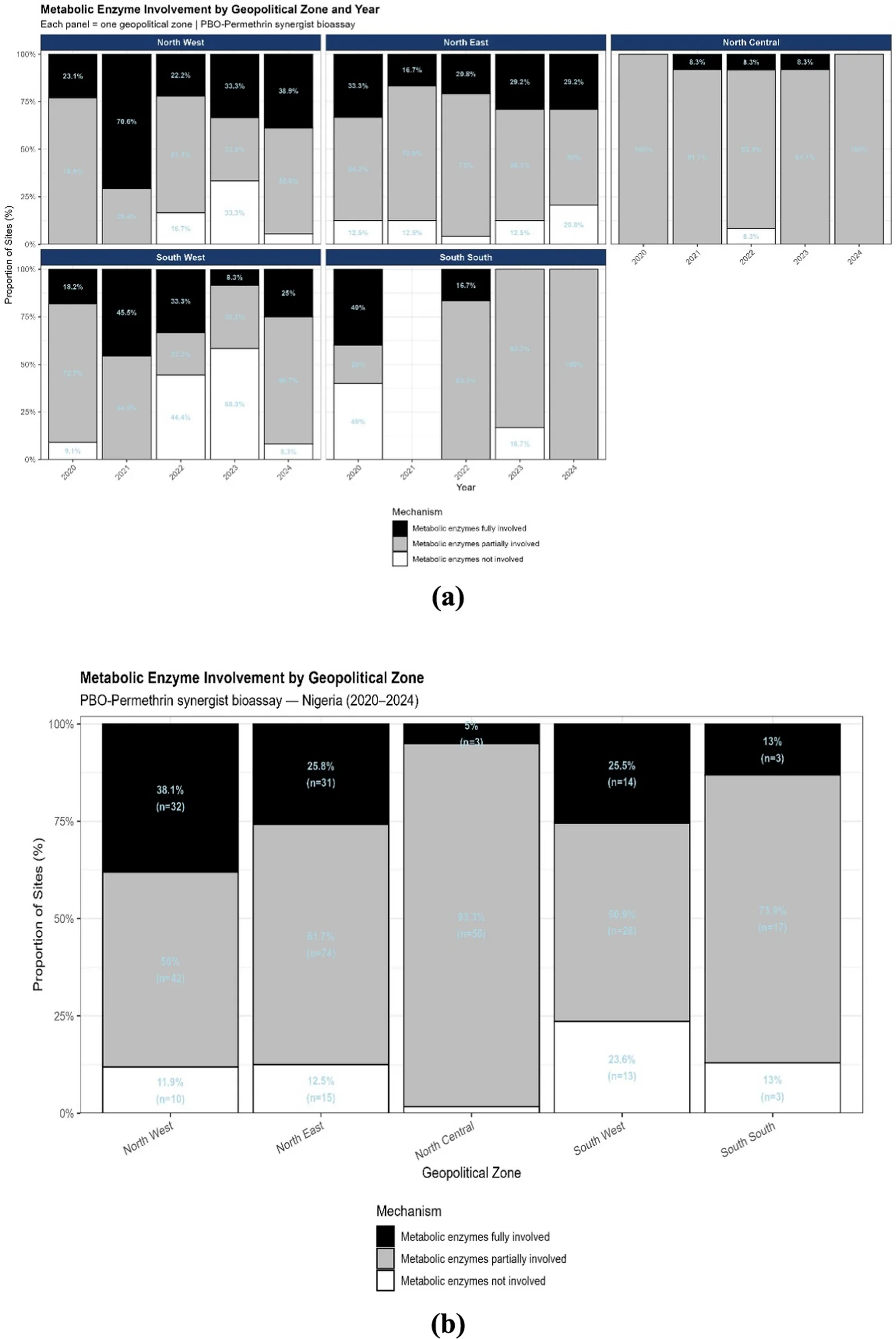
Overall contribution of metabolic resistance mechanisms in *Anopheles gambiae* s.l. across the geopolitical zones of Nigeria: **a** temporal trends from 2020 to 2024; and **b** overall distribution across geopolitical zones

Marked geographical heterogeneity was observed in the contribution of metabolic resistance mechanisms. Complete restoration of susceptibility following PBO exposure was most frequent in Katsina (66.7%), Yobe (53.3%), Osun (42.3%), and Taraba (30.0%), whereas lower proportions were recorded in Ogun (10.3%), Kwara (10.0%) and Delta (13.0%) (Fig. 8). At the geopolitical level, complete metabolic enzyme involvement was highest in the Northwest (38.1%) and lowest in the North Central zone (5.0%), where resistance was almost exclusively attributable to partial metabolic enzyme involvement (93.3%) (Fig. 9). Temporal trends varied across regions, although partial metabolic enzyme involvement remained the dominant mechanism throughout the surveillance period (Fig. 9).

### Spatial and temporal distribution of the *kdr-w* mutation frequency in malaria vectors

**Fig. 10:**
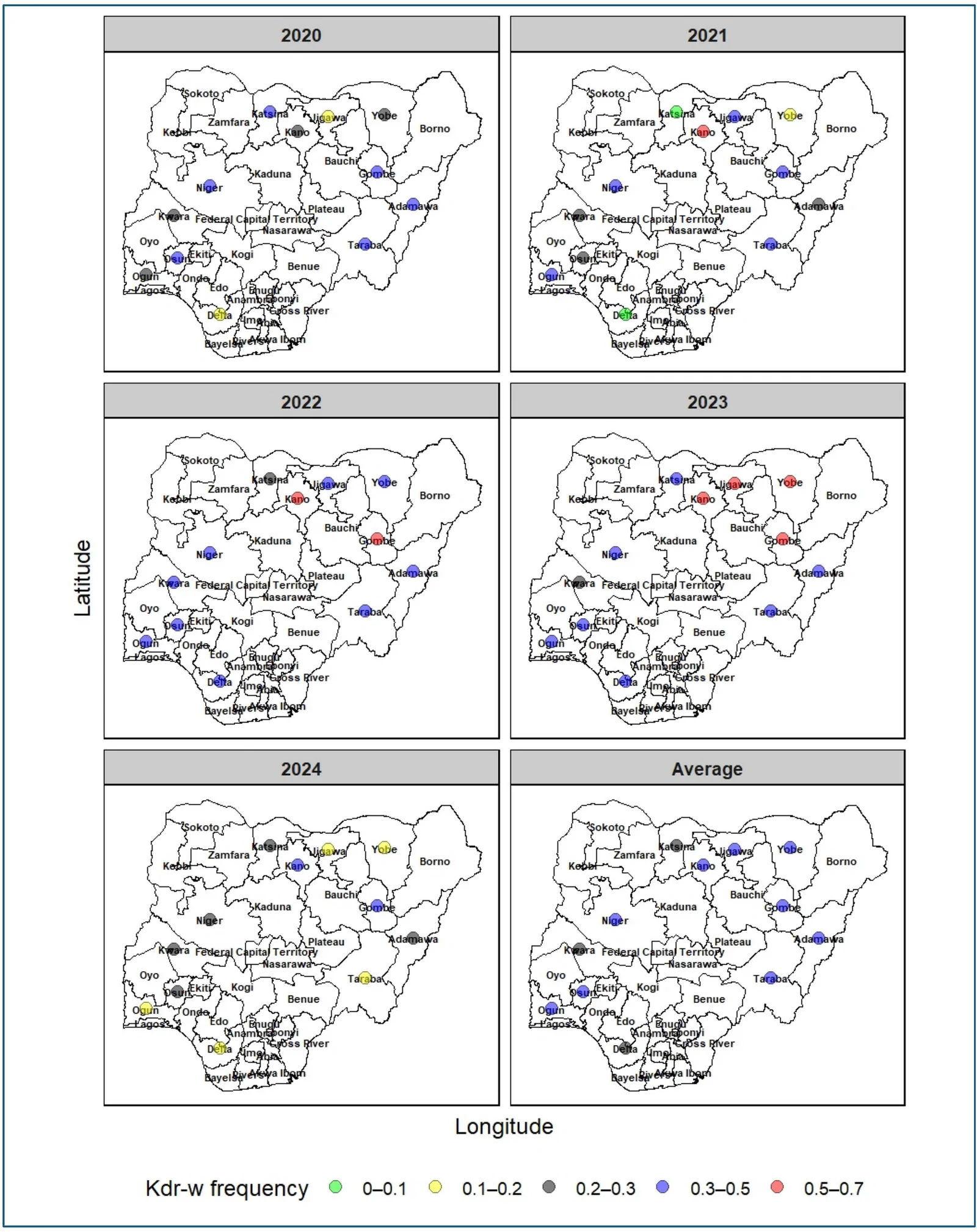
Spatial and temporal distribution of the *kdr-w* mutation frequency in malaria vectors across selected Nigerian states from 2020 to 2024

Spatial and temporal distribution of the *kdr-w* mutation frequency in malaria vectors across selected sites from 2020 to 2024, including the 5-year average is presented in Fig. 10. Across the study period, *kdr-w* frequencies were predominantly low to moderate (0.3–0.5) in most locations. However, consistently higher frequencies (0.5–0.7) were observed in several northern areas, notably Jigawa, Kano, and Katsina States, with occasional high-frequency occurrences in parts of Taraba and Yobe in subsequent years. Although year-to-year variation was evident at some sites, there was no clear nationwide increasing trend. The 5-year average map indicates persistent spatial clustering of elevated *kdr-w* frequencies in these northern locations, suggesting sustained selection pressure for target-site resistance (Fig. 10).

### Association between *kdr-w* frequency and mosquito mortality

Kendall’s tau correlation analysis revealed insecticide-specific associations between *kdr-w* mutation frequency and mosquito mortality to pyrethroid insecticides. A significant negative association was observed for deltamethrin (τ =  − 0.179, p < 0.001), indicating reduced mortality at higher *kdr-w* frequencies (Table 2). For alphacypermethrin, a weak negative association was detected that approached statistical significance (τ =  − 0.069, p = 0.055). In contrast, no association was observed between *kdr-w* frequency and mortality to permethrin (τ ≈ 0, p = 0.998). These findings suggest that target-site resistance mediated by the *kdr-w* mutation contributes more strongly to deltamethrin resistance than to resistance against the other pyrethroids assessed (Table 2).

**Table 2:** Kendall’s tau correlation analysis revealing insecticide-specific associations between *kdr-w* mutation frequency and mosquito mortality to pyrethroid insecticides

Insecticide	Tau	p-value
Alphacypermethrin	−0.0692	0.0548
Deltamethrin	−0.179	7.17 × 10^–8^
Permethrin	−0.0000948	0.998

A linear mixed-effects model was used to examine the relationship between *kdr*-w frequency and mosquito mortality following pyrethroid exposure while accounting for insecticide type, survey year, and the hierarchical structure of the surveillance data (Table 3). After adjustment for insecticide type and year, *kdr*-w frequency was not associated with mosquito mortality (β = 1.61, *P* = 0.854), indicating that variation in target-site mutation frequency alone did not explain the observed differences in pyrethroid susceptibility. By contrast, mortality remained significantly higher following exposure to deltamethrin than to alphacypermethrin (β = 8.75, *P* < 0.001), whereas no significant difference was observed between permethrin and alphacypermethrin (β = 2.77, *P* = 0.104). Survey year contributed significantly to overall model fit, although only a marginal increase in mortality was observed in 2023 relative to 2020 (β = 5.14, *P* = 0.052).

**Table 3:** Fixed effects estimates from the linear mixed-effects model assessing factors associated with mosquito mortality to pyrethroids

Predictor	Estimate (β)	Std. Error	df	t value	p value
Intercept	37.95	6.64	17.95	5.72	< 0.001
kdr-w frequency	1.61	8.75	1011.51	0.18	0.854
Deltamethrin	8.75	1.7	1000.96	5.15	< 0.001
Permethrin	2.77	1.7	1000.96	1.63	0.104
Year (2021 vs 2020)	−2.27	2.19	1000.97	−1.03	0.302
Year (2022 vs 2020)	−2.43	2.35	1003.06	−1.04	0.301
Year (2023 vs 2020)	5.14	2.64	1005.69	1.95	0.052
Year (2024 vs 2020)	0.15	2.3	1002.48	0.07	0.948

Random-effects estimates revealed substantial variation in mosquito mortality among states (variance = 405.79), whereas variation among LGAs within states was comparatively small (variance = 1.98) (Table 4). This finding suggests that resistance patterns are more strongly structured at the state level than among LGAs within the same state, potentially reflecting differences in insecticide use, vector control interventions, or the distribution of resistance mechanisms.

**Table 4:** Random effects variance components from the linear mixed-effects model

Random effect	Variance	Std. Dev
State (intercept)	405.79	20.14
LGA within state (intercept)	1.98	1.41
Residual	519.75	22.80

The mixed-effects model showed that the fixed effects (kdr-w frequency, insecticide type and survey year) explained only 2.2% of the observed variation in mosquito mortality (marginal R^2^ = 0.022), whereas incorporating the hierarchical spatial structure increased the explained variation to 45.2% (conditional R^2^ = 0.452) (Table 5). These findings indicate that temporal variation, insecticide type and kdr-w frequency together accounted for only a small proportion of the observed variation in pyrethroid susceptibility. Instead, resistance patterns were predominantly driven by spatial heterogeneity among states and LGAs, supporting an important contribution of local ecological conditions, insecticide exposure history and additional resistance mechanisms, particularly metabolic resistance.

**Table 5:** Model fit and variance explained by the linear mixed-effects model assessing mosquito mortality

Metric	Value
Marginal R^2^ (fixed effects)	0.022
Conditional R^2^ (fixed + random effects)	0.452
Observations	1080
States	12
LGAs	72

## Discussion

This nationwide surveillance provides a comprehensive assessment of temporal changes in malaria vector species composition, insecticide susceptibility, and resistance mechanisms across Nigeria over a 5-year period. The findings demonstrate an increasing predominance of malaria vectors, widespread resistance to pyrethroid insecticides, sustained susceptibility to pirimiphos-methyl, and evidence that metabolic resistance, rather than target-site resistance alone, might be the principal mechanism underlying pyrethroid resistance across much of the country.

### Species composition and epidemiological implications

The increasing predominance of *An*. *coluzzii* observed during the surveillance period suggests a temporal shift in the composition of the *Anopheles gambiae* complex. Although collections were restricted to the rainy season, the standardized sampling protocol and repeated surveillance at the same sentinel sites each year reduce the likelihood that this pattern is attributable solely to seasonal variation. The observed trend is further supported by independent longitudinal data from Osun State [3], where *An*. *coluzzii* similarly predominated over a 2-year surveillance period. Similar shifts have been reported elsewhere in West Africa, where urbanization, irrigation expansion and year-round larval habitats favour *An*. coluzzii over *An*. *gambiae* s.s. and *An*. *arabiensis* [23–25]. Because these sibling species differ in host preference, resting behaviour and responsiveness to vector control interventions [2, 16], continued expansion of *An*. *coluzzii* may influence local malaria transmission dynamics and the effectiveness of existing vector control strategies. The predominance of *An*. coluzzii is particularly important because it is an efficient vector of *Plasmodium falciparum*, *P*. *malariae* and *P. ovale* [26, 27].

### Insecticide resistance trends and operational implications

The widespread and persistent resistance to permethrin, deltamethrin and alphacypermethrin demonstrates that pyrethroid resistance is now well established across the surveyed states. Similar resistance profiles have been reported in Nigeria and neighbouring countries [9–14], indicating that pyrethroid resistance is widespread throughout the region. Although localized pockets of susceptibility persisted in a few LGAs, mortality remained consistently low for permethrin and alphacypermethrin across most locations, suggesting that pyrethroid-only long-lasting insecticidal nets (LLINs) are likely to provide reduced operational effectiveness in many settings. Small year-to-year fluctuations in susceptibility were observed in a limited number of sentinel sites, with some populations alternating between resistant and susceptible phenotypes. These localized changes likely reflect the potential impact of temporal variation in mosquito population composition, insecticide selection pressure, ecological conditions, and fluctuations in resistance allele frequencies rather than sustained reversals of resistance. Importantly, these changes were geographically restricted and did not alter the overall pattern of widespread pyrethroid resistance observed throughout the surveillance period. Among the three pyrethroids evaluated, permethrin consistently exhibited the lowest mortality, whereas deltamethrin retained comparatively greater bio-efficacy. Collectively, these findings support continued implementation of insecticide resistance management strategies, including insecticide rotation and the deployment of dual-active ingredient vector control tools to reduce selection pressure on pyrethroids [11, 18].

In contrast, susceptibility to pirimiphos-methyl remained high across most surveillance sites throughout the study period. The few localized reductions in mortality observed in Yobe, Kano and Niger between 2022 and 2023 were limited in geographic extent and did not indicate widespread organophosphate resistance. These findings support the continued use of pirimiphos-methyl for indoor residual spraying while reinforcing the importance of routine resistance surveillance and insecticide rotation to preserve its effectiveness in accordance with WHO recommendations [18].

### Resistance mechanisms and their contribution to pyrethroid resistance

Our findings indicate that pyrethroid resistance in Nigerian malaria vectors is mediated by multiple mechanisms, with metabolic resistance emerging as the predominant contributor, particularly for permethrin resistance. Synergist bioassays showed complete or partial restoration of susceptibility in most resistant mosquito populations, consistent with an important role for cytochrome P450-mediated detoxification. This interpretation is supported by previous studies from Nigeria, which have consistently identified elevated metabolic enzyme activity as a major determinant of pyrethroid resistance, particularly in Anopheles coluzzii [28, 29]. The increasing predominance of An. coluzzii observed during the surveillance period may therefore contribute to the widespread metabolic resistance detected in this study.

The predominance of metabolic resistance has important implications for malaria vector control. Cytochrome P450 enzymes have been associated not only with resistance to pyrethroids but also with reduced susceptibility to other insecticide classes, raising concerns about the potential evolution of cross-resistance as new vector control tools are introduced [30]. Consequently, the widespread metabolic resistance documented here provides additional support for the deployment of dual-active ingredient LLINs, including Interceptor^®^ G2, which combines chlorfenapyr with alpha-cypermethrin and has demonstrated improved epidemiological and entomological performance over pyrethroid-only LLINs in areas with established pyrethroid resistance [32]. Although chlorfenapyr and clothianidin susceptibility was not evaluated in the present study, increasing deployment of these insecticides may alter selection pressures acting on vector populations. Recent studies also suggest that metabolic mechanisms may influence susceptibility to newer insecticide classes, including the neonicotinoid clothianidin and, under certain conditions, the pyrrole chlorfenapyr, although the underlying mechanisms and their operational significance remain under investigation [30–32]. Continued surveillance will therefore be essential to detect early changes in susceptibility as these interventions become more widely deployed.

The mixed-effects analysis further demonstrated that kdr-w frequency was not independently associated with mosquito mortality after accounting for insecticide type and temporal variation. Instead, most of the explained variation was attributable to differences among states and LGAs, indicating that local ecological conditions, insecticide exposure histories and additional resistance mechanisms contribute more substantially to pyrethroid resistance than target-site mutations alone. This interpretation is consistent with the synergist bioassay findings, which identified metabolic resistance as the principal mechanism underlying pyrethroid resistance across the surveyed populations.

The observed geographic heterogeneity in both metabolic resistance and elevated kdr-w frequencies is likely to reflect differences in local selection pressures. Northern Nigeria has experienced substantial expansion of irrigated agriculture, particularly rice cultivation, accompanied by widespread use of agricultural insecticides. These practices have previously been implicated in selecting for resistance through cross-selection between agricultural and public-health insecticides [12–14]. Although agricultural pesticide use was not quantified directly in this study, strengthening collaboration between the health and agricultural sectors, improving pesticide stewardship and expanding integrated vector management are likely to reduce resistance selection pressure. Future surveillance should also integrate standardized monitoring of agricultural pesticide use, including application practices and insecticide residue assessments, alongside routine entomological surveillance to better quantify the contribution of agricultural selection pressure to insecticide resistance.

Finally, as Nigeria expands the deployment of dual-active ingredient LLINs and other novel insecticide-based interventions, routine resistance surveillance should extend beyond phenotypic susceptibility testing to include molecular, biochemical and synergist-based characterization of resistance mechanisms. Such an integrated surveillance framework would facilitate early detection of emerging resistance, improve understanding of evolving resistance pathways and provide evidence to support timely insecticide resistance management decisions.

### Implications for malaria vector control

The findings of this study support the need to diversify malaria vector control strategies in Nigeria. Given the widespread resistance to pyrethroid insecticides, greater reliance on non-pyrethroid interventions, including pirimiphos-methyl-based IRS and dual-active ingredient LLINs, should be considered where appropriate. Complementary approaches such as larval source management in suitable ecological settings, biological control, and emerging genetic technologies, including gene-drive systems, may further reduce dependence on conventional insecticides [33, 34]. Successful implementation of these interventions will require continued surveillance, careful ecological evaluation, and appropriate regulatory oversight.

Overall, the increasing predominance of *An*. *coluzzii*, widespread pyrethroid resistance, persistent hotspots of target-site resistance, and the dominant contribution of metabolic resistance underscore the need for adaptive insecticide resistance management strategies in Nigeria. Sustained nationwide surveillance integrating phenotypic, molecular and synergist bioassays will be essential for guiding evidence-based decisions on insecticide deployment and maintaining the effectiveness of malaria vector control interventions.

### Limitations

This study has several limitations that should be acknowledged. First, although surveillance was conducted across 12 states and 72 LGAs spanning Nigeria's major ecological zones, it may not fully capture the heterogeneity of malaria vector species composition and insecticide resistance patterns nationwide. Second, mosquito collections were undertaken during the rainy season (April–October), when vector densities are highest and routine national surveillance is conducted. Consequently, the study does not capture potential seasonal variation in vector abundance, species composition, or insecticide resistance that may occur during the dry season. Although year-round surveillance would provide a more comprehensive understanding of temporal changes in resistance dynamics, the large geographic coverage and 5-year duration of this study provide a robust assessment of resistance patterns during the principal malaria transmission season in Nigeria.

Third, although kdr-w genotyping and synergist bioassays provided important insights into target-site and metabolic resistance mechanisms, more detailed molecular and biochemical investigations, including *CYP450, CYP6P3, CYP6M2 and GSTe2* expression profiling and enzyme activity assays, were not undertaken and would have enabled a more comprehensive characterization of metabolic resistance pathways. Fourth, molecular analyses were limited to the *kdr-w (L995F)* mutation. Other target-site mutations, including *kdr-e (L995S)*, together with additional resistance-associated genetic markers, were not investigated and may also contribute to the observed resistance phenotypes. Finally, although agricultural pesticide use is a plausible driver of resistance selection, direct measurements of agrochemical exposure and application practices were not available, limiting our ability to quantify their contribution to resistance development.

Finally, insecticide susceptibility was assessed using WHO diagnostic-dose bioassays, which classify mosquito populations based on a single diagnostic threshold. Consequently, localized year-to-year changes in phenotypic status may reflect fluctuations around this threshold rather than true reversals of resistance. Although such changes were uncommon and did not alter the overall pattern of widespread pyrethroid resistance observed across the surveillance period, incorporating resistance intensity bioassays (5 × and 10 × diagnostic concentrations) into future surveillance would provide a more comprehensive assessment of resistance strength and improve interpretation of temporal changes in susceptibility.

## Conclusions

This 5-year nationwide surveillance demonstrates important changes in malaria vector populations in Nigeria, characterized by the increasing predominance of *Anopheles coluzzii* and widespread pyrethroid resistance may be driven primarily by metabolic detoxification mechanisms, with localized contributions from target-site (kdr-w) mutations. Although kdr-w was associated with insecticide-specific variation in mortality, particularly for deltamethrin, much of the observed heterogeneity in resistance occurred at the state and LGA levels, emphasizing the importance of local ecological and operational factors. While pirimiphos-methyl remains largely effective, emerging pockets of reduced susceptibility highlight the need for continued monitoring to preserve its efficacy. These findings support strengthening resistance management through routine nationwide surveillance that integrates phenotypic, molecular and metabolic resistance monitoring, the deployment of next-generation LLINs incorporating synergists or dual active ingredients in areas with high metabolic resistance, and evidence-based rotation of insecticides for indoor residual spraying in accordance with local resistance profiles. Sustained investment in integrated vector management and geographically tailored resistance monitoring will be essential to maintain the effectiveness of current interventions and support progress towards malaria elimination in Nigeria.

## Supplementary Information

Supplementary material 1.

## Data Availability

All data generated and analyzed during this study are included in this published article. The raw datasets are available from the corresponding author upon reasonable request.

## Abbreviations

LLIN: Long-lasting insecticidal net
IRS: Indoor residual spraying
EIR: Entomological inoculation rate
Kdr: Knockdown resistance
LGA: Local Government Area
WHO: World Health Organization
