# Supplementary material for: Insecticide resistance profile of the Anopheles gambiae s.l. mosquitoes in Nigeria: a 5-year malaria vector surveillance report
Source: Malar J. 2026 Aug 10;25:404. doi: 10.1186/s12936-026-06083-9 (PMC13741510; doi:10.1186/s12936-026-06083-9)
Supplement: Supplementary file 1 [file 12936_2026_6083_MOESM1_ESM.pdf]

Insecticide Resistance profile of members of the *Anopheles gambiae* s.l. mosquitoes in Nigeria: a five-year malaria vector surveillance report

Adedapo O. Adeogun *[1], Ayodele S. Babalola *[1], Tolulope A. Oyeniyi[1], Okoko O. Oyale[2], Romoke T. Izekor[1], Oluwakemi Adetunji[1], Abiodun Olakiigbe[1], Olalekan Olagundoye[1], Julius A. Bamidele[3], Adetunji I. Adeniran[3], Nofisat F. Lawal[3], Philemon Babylon[4], Atinga Atimi[5], Sufi A. Danjuma[6], Tasiu Shuaibu[6], Jibrin M. Mabu[7], Kabiru Salisu[8], Asma'u H. Abubakar[9], Mahmud Sufyan[10], Kabir B. Abdullahi[11], Adamu Isa[12], Ezra Abba[13] Abdulmalik B. Shuaibu[13], Victor N. Enwemiwe[14], Esiwo Eric[14], Wama B. Emmanuel[15], Liatu C. Garba[15], Abdullahi M. Daskum[16], Monsuru A. Adeleke[17], Chioma C. Ojianwuna[14], Elkanah O. Sambo[15], Adedayo Oduola[18], Petrus U. Inyama[18], Lazarus Samdi[18], Abiodun Obembe[19], Mustapha M. Dogara[6], Kennedy P. Yoriyo[13], Suleiman Mohammed[11], Rebecca S. Naphtali[20], Chioma Amajoh[21], Adesoye Oluwaseun[22], Fatima Ali[2], Adesola Musa[1], Wahedi Jasini[23], Bala Mohammed[2], Esema Mary[2], Omo-Eboh Mamudu[2], Olufunmilayo A. Idowu[3], Adeolu Ande[24], Kayode I. Olayemi[25], Manu Yayo Abdulsalami[26], Ademu Cyril[2], Okoronko Chukwu[2], Ozor Lynda[27], Ssekitooleko James[28], Kawu Issa[28], Mokuolu Olugbenga[29], Nnenna Ogbulafor[2], Awolola Samson[1]

Supplementary File S1

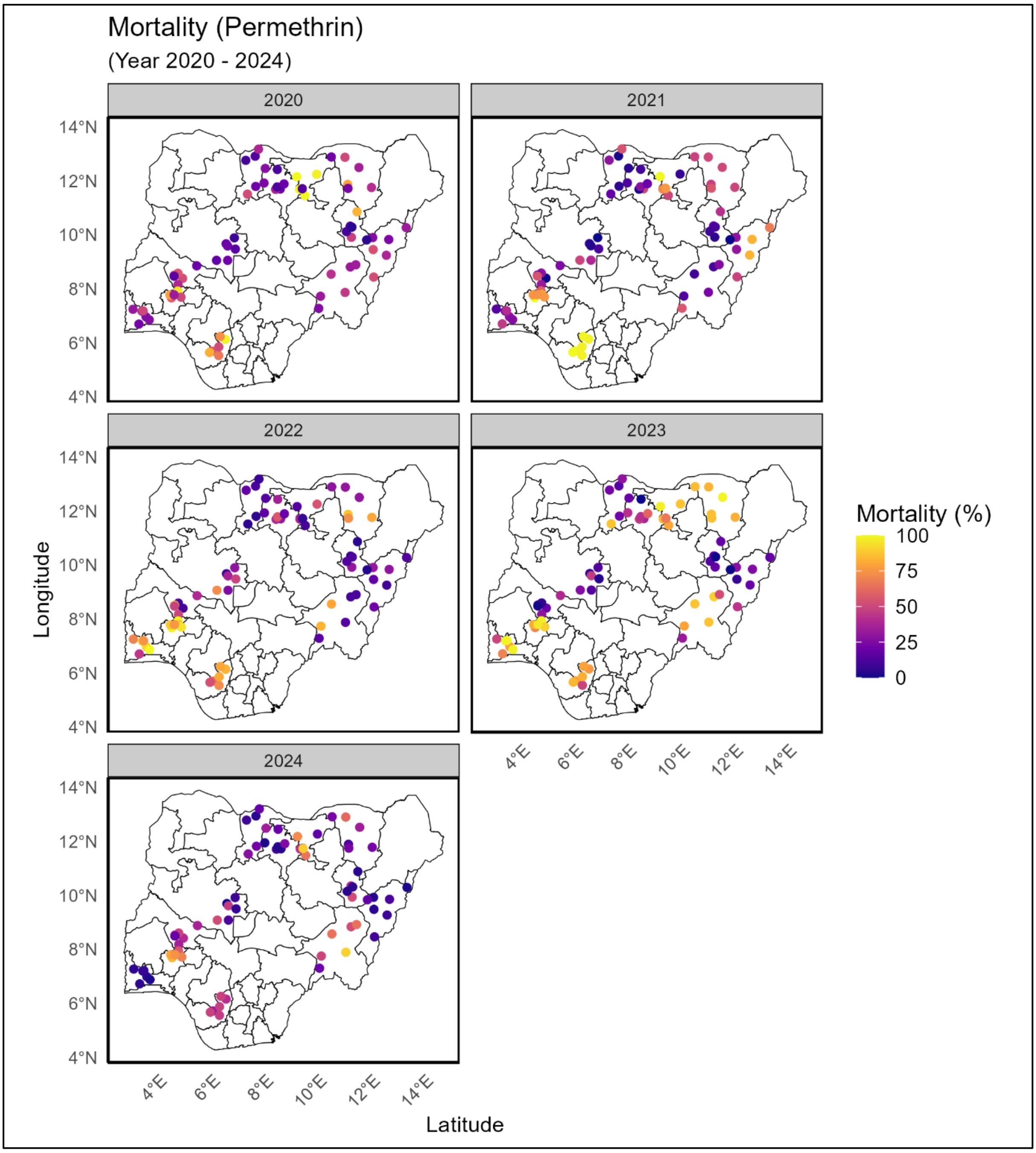

Figure A: Percentage mortality of An. gambiae s.l. exposed to the diagnostic concentration of permethrin (WHO tube bioassay) in 72 Local Government Areas across 12 states in Nigeria from 2020 to 2024

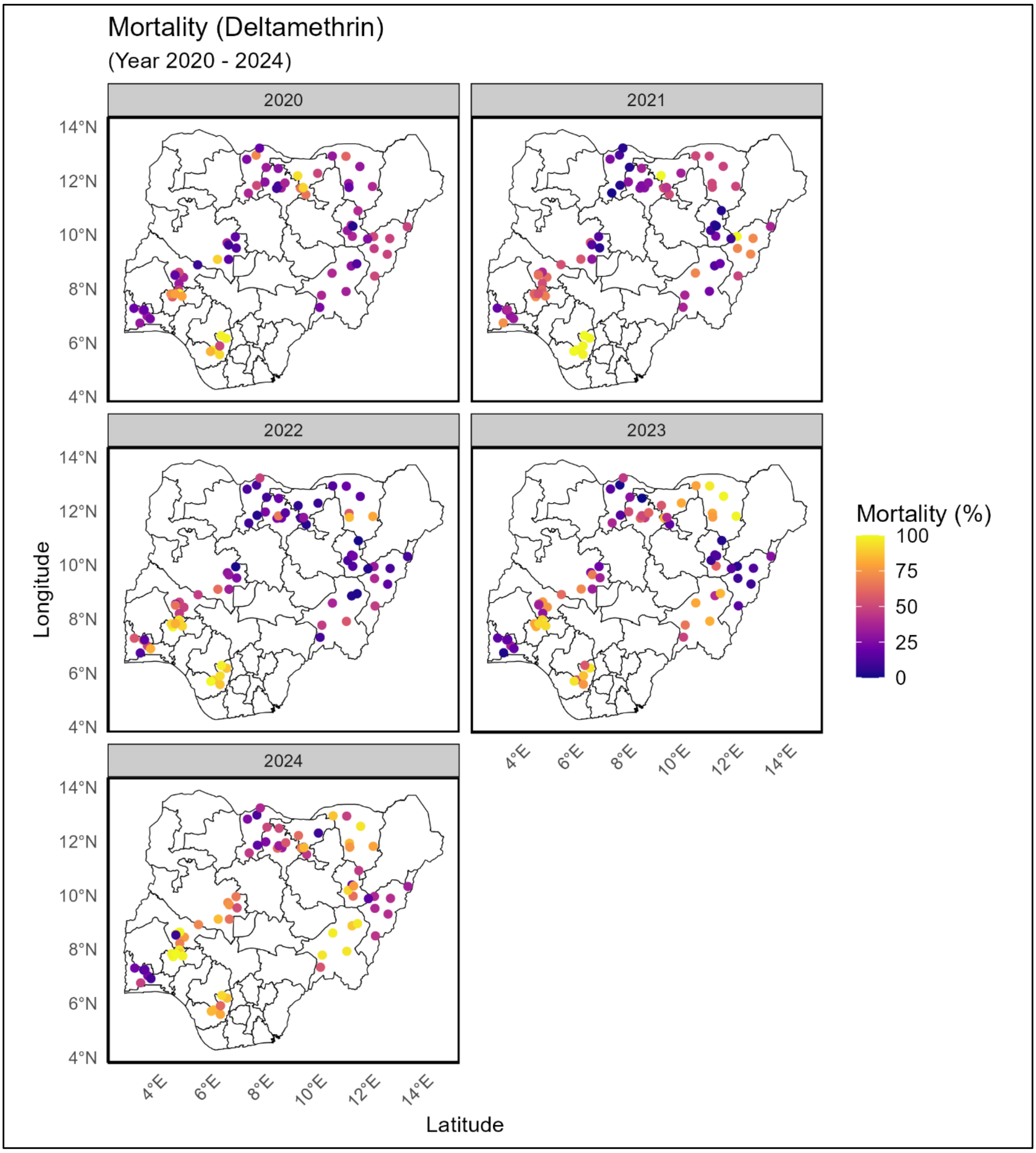

Figure B: Percentage mortality of An. gambiae s.l. exposed to the diagnostic concentration of deltamethrin (WHO tube bioassay) in 72 Local Government Areas across 12 states in Nigeria from 2020 to 2024

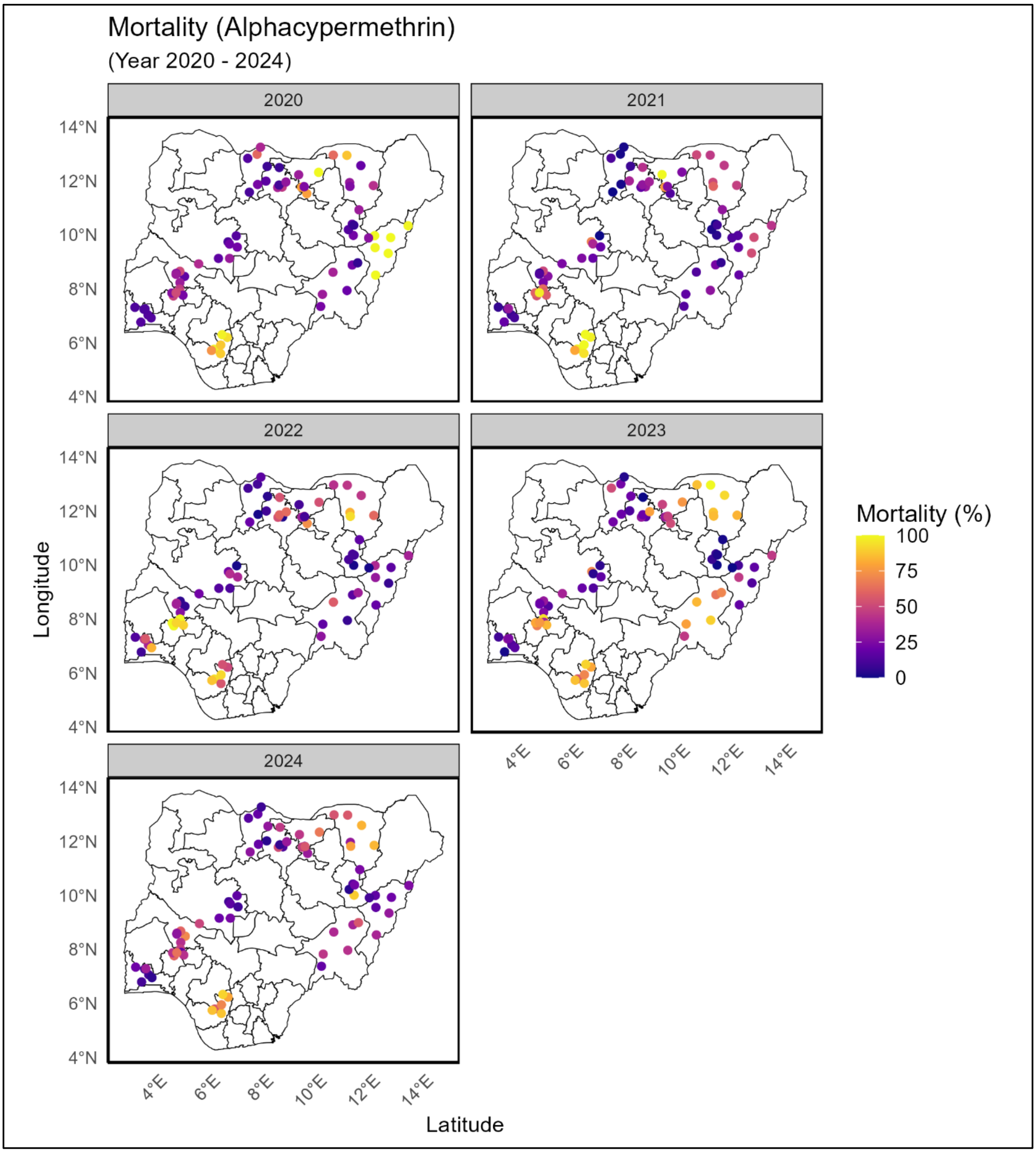

Figure C: Percentage mortality of An. gambiae s.l. exposed to the diagnostic concentration of alphacypermethrin (WHO tube bioassay) in 72 Local Government Areas across 12 states in Nigeria from 2020 to 2024

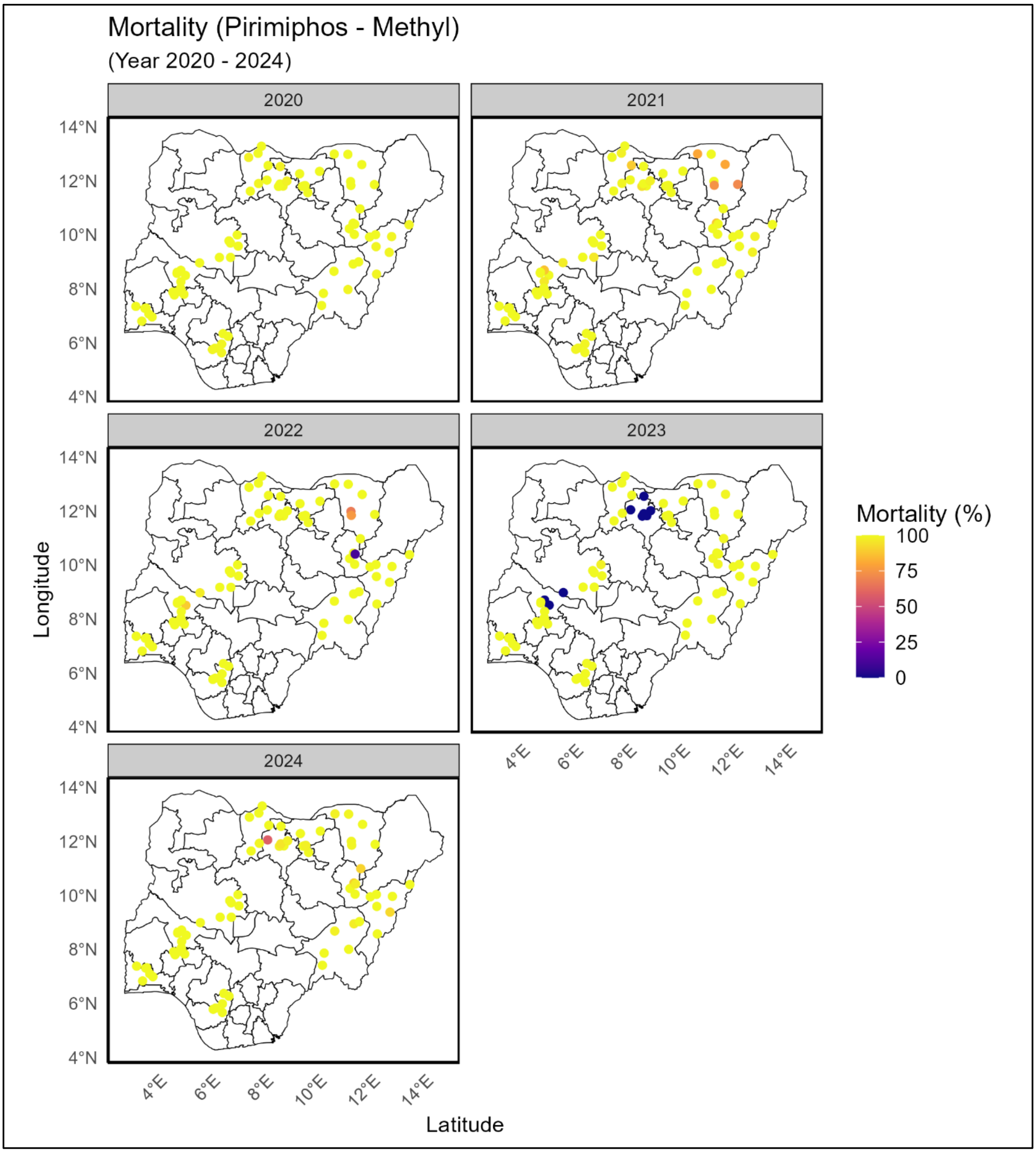

Figure D: Percentage mortality of An. gambiae s.l. exposed to the diagnostic concentration of pirimiphos-methyl (WHO tube bioassay) in 72 Local Government Areas across 12 states in Nigeria from 2020 to 2024
